# Towards a more individualised assessment of post-training fatigue in young footballers

**DOI:** 10.1016/j.heliyon.2024.e33626

**Published:** 2024-06-27

**Authors:** Adrien Mangini, Robin Macchi, Dorian Giraud, Laura Pomportes, Paul Galantine, Denis Bertin, Caroline Nicol, Arnaud Hays

**Affiliations:** a Aix-Marseille Univ, CNRS, HIPE Human-Lab (UAR 202324378), Marseille, France; b Aix-Marseille Univ, CNRS, ISM (UMR 7287), Marseille, France; c French Institute of Sport (INSEP), Laboratory Sport, Expertise and Performance (EA 7370), Paris, France; d Aix-Marseille Univ, CNRS, IUSTI (UMR 7343), Marseille, France; e Toulon Univ, J-AP2S (UR 201723207F), Toulon, France

**Keywords:** Force-velocity profile, Fatigue, Drop jump, Training individualisation, Young athletes

## Abstract

Despite improvements in training load and fatigue monitoring, fatigue status may adversely affect intrinsic risk factors, particularly in young footballers. This study aimed to better individualise the fatigue effects of a 75-min football training session in young elite male players.

Eighteen players (15.6 ± 1.7 years) completed a test battery before and after a football training session. Assessments included capillary blood samples (HCO_3_^−^, lactate, pH), subjective ratings of muscle soreness and fatigue. Functional tests included 30 m field sprint, laboratory drop jump (DJ) and horizontal force-velocity (H-FvP). The k-means method was applied to the first two dimensions of principal component analysis of changes in the H-FvP and DJ tests.

Football training resulted in significant physiological changes and functional impairments, in particular an increase in interlimb asymmetry in the DJ test. No significant fatigue effect was found on the H-FvP test data. However, confirming the interest of combining the two tests, cluster analysis revealed two subgroups: In the H-FvP test, Cluster 1 decreased in $\bar{V}$_0_ (p < 0.001) and $\bar{P}$_max_ (p < 0.01), while Cluster 2 decreased in $\bar{F}$_0_ (p < 0.001). In the DJ test, Cluster 1 decreased in mean velocity (p < 0.01), relative mean and maximum power (p < 0.01) during push-off, while Cluster 2 decreased in relative mean push-off force (p < 0.01) and increased in interlimb asymmetry during braking (p < 0.01).

This study highlights the contribution and complementarity of the H-FvP and DJ tests to improve individual screening for fatigue-related functional changes in footballers. Extrapolated values from the H-FvP test led to the identification of two subgroups with opposite fatigue profiles. One subgroup showed increased interlimb asymmetry in DJ, indicating an increased risk of injury with fatigue. These findings highlight the need for individualised fatigue assessment in young footballers.

## 1 Introduction

Several studies have highlighted the interaction between performance, fatigue and recovery in football [[1], [2], [3]]. Despite recent improvements in training load and fatigue monitoring, fatigue status may adversely affect intrinsic risk factors and alter the injury risk profile of both professional and young players [4]. Musculoskeletal injuries occur frequently in non-contact situations [5], with a higher incidence observed at the end of each half [6]. Although less studied, they also occur during training [7]. Due to physical immaturity, accelerated growth, young players would have greater inter-individual movement variability and more pronounced interlimb asymmetry during jumping [8,9], making them more prone to injury during training than adults [10]. In a large cohort of 357 elite young footballers, greater landing force asymmetry was even the only risk factor significantly associated with an increased lower limb injury risk [9].

Both field and laboratory test batteries are increasingly used to assess the fitness of football players, identify injury risk and quantify interlimb asymmetries [4,9]. Multi-joint sprint and vertical jump tests are commonly used to quantify functional fatigue effects [11,12] and to predict injury-risk [13]. In particular, the Drop Jump (DJ) test can reveal a reduced tolerance to ground impact [14]. In this test, the reactive strength index (RSI), which represents the ratio of jump height to contact time, is considered to be a significant predictor of injury and is sensitive to fatigue status [15]. As highlighted in the review by Bishop et al. (2018), interlimb asymmetry is highly individual in nature, and at that time there was a lack of data under fatiguing conditions and quantifying its changes with fatigue to further understand injury mechanisms. More recently, quantifying and monitoring fatigue-related changes in performance, RSI and interlimb asymmetry in DJ has been considered of interest to detect potential changes in the injury risk profile after a football match [13,15,16]. These variables have also been used to guide return to sport decisions [17]. However, due to the high task dependency in response to a football match [18], there is a need to clarify the underlying individual functional deficits.

Force-velocity-power (FvP) profiling is of particular interest for differentiating inter-individual responses and identifying training and potential fatigue effects [19]. In footballers, this simple field method has been used to model a linear FvP profiling based on vertical squat jumps at varying loads [20] or repeated sprints [21]. However, loaded vertical jumps may be inadvisable in adolescents due to the prevalence of poor squatting and landing technique [22], and low back strain [23]. Conversely, the horizontal type of force-velocity-power jump (H-FvP) test, performed under laboratory conditions, has demonstrated its relevance in such a young population [24] and its interest in differentiating inter-individual responses to fatigue [25].

This study combined the DJ and H-FvP tests to better assess the individual fatigue effects of a standard 75-min football training session on lower limb muscle function in elite young male players. The test battery included a series of assessments including metabolic and perceptual responses, a 30 m field sprint, a DJ and H-FvP tests in the laboratory. Our first hypothesis was that a football training session would result in significant functional impairment, particularly in the DJ test. The second hypothesis was that clustering analysis based on functional changes would be useful to identify subgroups of young players with specific signs of fatigue.

## 2 Material and methods

### 2.1 Participants

Eighteen young elite male players from a football training centre participated in the study (age: 15.6 ± 1.7 years; height: 173.6 ± 8.4 cm; body mass: 62.1 ± 11.4 kg). Recruitment was carried out internally by the management team of the training centre. Only players free of musculotendinous and/or osteoarticular injuries for at least 3 months were included. The study group consisted of 9 defenders, 5 midfielders and 4 fowards. The players' training status was classified as ‘high’, with more than 8 h/5 training sessions per week and one competitive match [3]. The dominant lower limb was the limb preferentially used to kick a ball. Of the 18 participants, 13 had a dominant right leg and 5 had a dominant left leg. A total of 6 reserve players completed the test group when required for the 4v4 match situation without performing the experimental tests. The present study was approved by the National Ethics Committee (CERSTAPS n°IRB00012476-2021-13-04-106) and written informed consent was obtained from all players and their parents.

### 2.2 Experimental design

**Fig. 1:**
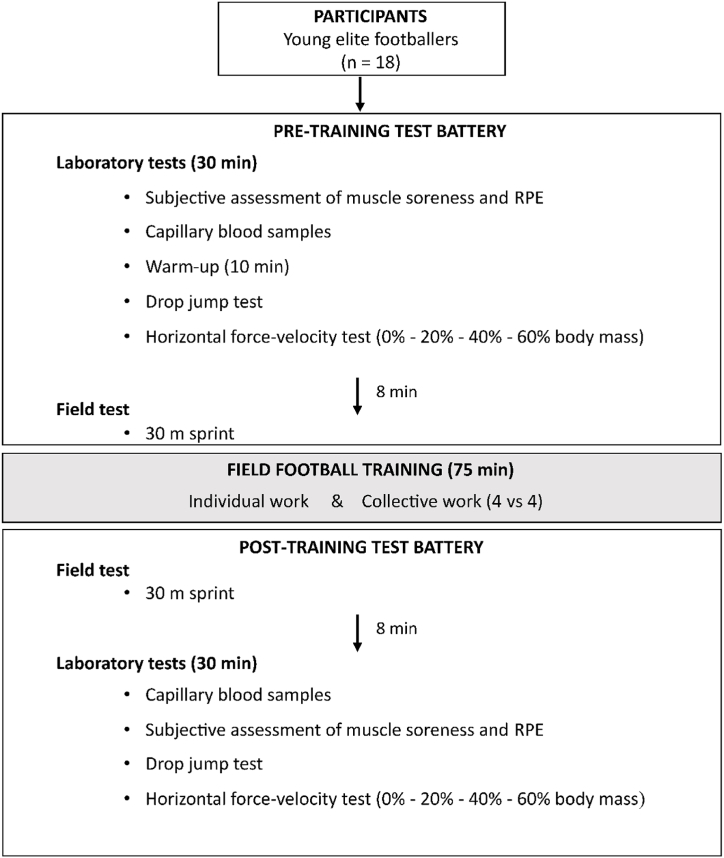
Experimental protocol. It included 2 test sessions, performed before (PRE) and after (POST) a regular football training session. RPE: Rate of perceived exertion.

A battery of tests, including a 30 m field sprint and laboratory drop jump (DJ) and horizontal force-velocity (H-FvP) tests, was performed before (PRE) and after (POST) a regular 75-min football training session (Fig. 1). All measurements were performed on two consecutive days, from 9 a.m. to 12 p.m., with one test day per player. Weather conditions were similar (mean 24 ± 1.2 °C) with no wind. Still water was available ad libitum.

The football training session was conducted by the club's physical trainers on an artificial turf pitch. The session started with a 30-min block of specific individual work. This was followed by a 45-min block of collective work in a match situation (4v4) with four mini-goals (no goalkeeper), consisting of 6 games of 5 min each, separated by 3 min of active rest. The dimensions of the field were 30 x 24, 720 m^2^. A 30 m sprint test was performed immediately before and after the training session.

At PRE and POST, laboratory testing began with an assessment of subjective muscle soreness and perceived global fatigue. Resting capillary blood samples were taken by the club physician to measure pH, bicarbonate ions (HCO_3_^−^) and lactate (the latter only at POST). At PRE, a 10-min warm-up was performed on a cycle ergometer (WattBike), followed by a 5-min rest before the DJ and H-FvP tests. Jumps were performed without warm-up at POST. The distance of 300 m between the laboratory and the football pitch was covered at a moderate running pace. At POST, the average time to start the laboratory tests was 8 min.

### 2.3 Experimental tests and measurements

On the field, the players were equipped with a tracking system (Polar Team 2, Polar Electro, Kempele, Finland, 100 Hz). The Global Positioning System (GPS) units were placed between the athlete's shoulder blades in a custom-made vest provided by the manufacturer. GPS derived locomotor performances were measured, including total distance, high speed running distance, distance covered at running speeds greater than 5.5 m s^−1^, total accelerations (>2 m s^−2^), total decelerations (>2 m s^−2^), sprint distance (distance covered at running speeds greater than 7.0 m s^−1^) and number of sprints [26].

The 30 m field sprint test consisted of two trials with a 30 s rest period. The start was made with both feet 0.5 m behind the first timing gate. The cells were positioned 1 m above the ground, which is approximately the height of the player's centre of mass [27]. The 30 m sprint time was measured using a wireless sports timing system (SmartSpeed, Fusion Sport, Australia) to an accuracy of 0.01 s.

Laboratory testing began with capillary blood collection by a physician using a safety lancet and a 95 μl heparinised capillary for pH and HCO_3_^−^ measurements (i-STAT clinical analyser, Abbott Point of Care, East Windsor, NJ, USA). Blood lactate concentration was quantified using a 5 μl heparinised capillary and Biosen C-line Clinic (EKF-Diagnostics GmbH, Germany).

Subjective ratings of involvement in the training session and muscle soreness were assessed using a visual analogue scale of 0–10. The rate of perceived exertion (RPE) on the Borg scale 6–20 was rescaled from 0 to 10 [28].

The jumping tests were performed in a randomised order as follows:

The DJ test consisted of three maximal trials from a drop height of 0.40 m, with 1 min of passive rest in between. Participants were instructed to keep their arms akimbo, look straight ahead at a visual cue on the wall, and were given verbal instructions to "rebound as quickly as possible with maximum effort immediately after ground contact" [29]. If all requirements were not met, the trial was repeated. Two force plates (Kistler 9287CA, 0.9 × 0.6 m) were used to record the vertical component of the ground reaction forces generated under each foot at a sampling frequency of 100 Hz.

The H-FvP test consisted of a series of squat jumps performed in the supine position on a frictionless sled (INPI deposit n° FR2011204) at four load levels: 0, 20, 40 and 60 % body mass (BM) [25]. Participants were instructed to apply force to the force plates as quickly as possible in order to reach maximum velocity at the end of the push-oﬀ. Countermovement was verbally forbidden and carefully controlled; the trial was repeated if necessary. A recovery period of 30 s was allowed between trials and 180 s between different loads. All trials were performed on two force plates (Kistler 9260AA3, 0.5 × 0.3 m, 2000 Hz). A linear encoder (Micro-Epsilon WDS-3000-P115-SR-U) attached to the sled measured the displacement to an accuracy of 0.1 cm. For each resistive load, the trial with the highest take-off velocity (vto) value was retained for post-processing.

### 2.4 Data processing

Data processing was performed using custom routines in Matlab (Matworks Inc, Novi, USA, R2019a).

For the DJ test, the best of the three trials (highest vto) was retained for statistical analysis. All calculations were based on the vertical component of the ground reaction force [20]. Contact time was divided into braking and push-off phases based on the time evolution of the velocity [30]. The analysis of the braking phase included its duration (T_Brake_), relative peak force (F_max/BM_), and relative mean force and power ($\bar{F}$_brake/BM_ and $\bar{P}$_brake/BM_, respectively). The analysis of the push-off phase included: vertical push-off time (Tpo), mean velocity ($\bar{V}$_po_), relative peak power output (P_max/BM_), and relative mean force and power ($\bar{F}$_po/BM_ and $\bar{P}$_po/BM,_ respectively) [31]. The RSI was calculated by dividing the jump height (m) by the contact time (s) [15]. To analyse the imbalance between the dominant (DL) and non-dominant (NDL) lower limb, the asymmetry index (LSI) was calculated for each kinetic variable of the DJ and H-FvP tests as follows [32]:

$$\text{LSI}=100\left(\;\frac{\left|\;\text{DL}-\text{NDL}\;\right|}{\text{mean}\;\left(\text{DL},\text{NDL}\right)}\;\right)$$

For the H-FvP test, a routine implemented in Matlab was used to detect the onset of push-off from the recorded force and displacement [25]. The push-off phase was analysed in a similar way to the DJ. A linear regression model was used to extrapolate the individual parameters of the FvP relationship: the intercepts ($\bar{V}$_0_ and $\bar{F}$_0_) and $\bar{P}$_max_ [33]. Each polynomial and linear relationship (excluding the extrapolated and $\bar{F}$_0_ and $\bar{V}$_0_ parameters) was evaluated by the coefficient of determination (R^2^), wich represents the variance explained by the model. The FvP relationships were fitted separately for the DL and NDL. Subsequent analysis focused on the highest recorded values of normalised peak force (F_max/BM_) achieved at 60 % BM loading and peak velocity (V_max_) achieved at 0 % BM loading. The push-off distance (H_po_), and the relative mean force ($\bar{F}$) and velocity ($\bar{V}$) were calculated at each load and normalised to BM. The LSI was calculated in a similar way to the DJ data.

### 2.5 Statistical analysis

Statistical analyses were performed using R v3.6.3 (R Core Team, 2020, R Foundation for Statistical Computing, Vienna, Austria). For each variable, a linear mixed model was fitted using the lmerTest package [34] and the lme4 package [35]. Session (PRE and POST) was included as a fixed effect and participant as a random effect. The significance of random factors was tested and the best model was selected using likelihood ratio tests of model comparisons with a backward selection method. Outliers were removed using the Grubbs test on residuals, and an ANOVA was then performed on the selected model. The normality of residuals was tested using the Shapiro-Wilk test. If normality was rejected, a mixed permutation model with approximately 10.000 permutations was used. Effect size was calculated using Cohen's d coefficient and assessed using the following thresholds: <0.2, 0.2 to <0.6, 0.6–1.2, and >1.2 for trivial, small, moderate, and large effects, respectively. All data are presented as mean ± standard deviation (SD) and as relative PRE-POST changes (delta %). Only significant changes are reported in the tables and figures.

Principal component analysis (PCA) was used to examine the relationships between the PRE-POST changes in the variables recorded in the H-FvP and DJ tests and to explore the individual responses. The variables were centred and reduced.

To assess the clustering trend and to identify fatigue profile subgroups, Hopkins statistics (i.e. H-value) was applied to the data from both tests [36]. If the H-value was significant, the k-means method was applied to the meaningful dimensions from the PCA analysis. The optimal number of clusters was determined using the "gap statistic method" (version 2.1.4, R).

## 3 Results

### 3.1 Football training session

The players covered a total distance of 6456 ± 849 m, including 224 ± 46 m at high-speed running. They performed 20 ± 4 sprints, 70 ± 7 accelerations, and 53 ± 15 decelerations during the 75-min football training session. The investment in the football training session was subjectively rated as 7.0 ± 0.2 on a scale of 0–10.

### 3.2 Acute field assessment of sprint performance

The 30 m sprint time showed a moderate increase (2 ± 2 %; p < 0.001; d = 0.72) at POST.

### 3.3 Perceptual responses (0–10 scale)

Subjective muscle soreness was low but showed a large increase after the training session (135 ± 52 %; from 1.4 ± 0.4 to 3.2 ± 0.7; p < 0.001; d = 1.8). There was also a large increase in the perceived level of fatigue (162 ± 36 %; from 2.4 ± 0.4 to 6.2 ± 0.6; p < 0.001; d = 2.41).

### 3.4 Metabolic responses

At POST, the capillary blood lactate concentration was 4.14 ± 0.26 mmol L^−1^. From PRE to POST, the pH decreased from 7.40 ± 0.01 to 7.38 ± 0.01 (p = 0.036; d = 0.56) and the HCO_3_^−^ concentration from 25.06 ± 0.39 to 21.58 ± 0.48 mmol L^−1^ (p < 0.001; d = 1.82).

### 3.5 Laboratory assessment of lower-limb muscle function

**Fig. 2:**
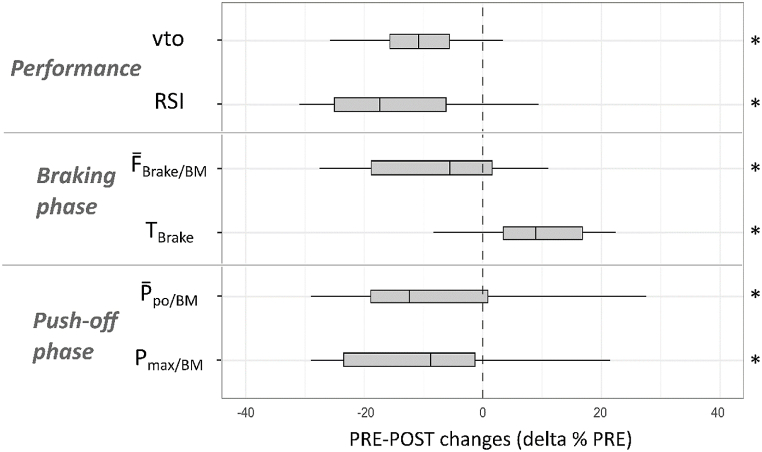
Relative PRE-POST changes (delta % PRE) in the DJ test. *p < 0.05. Take-off velocity (vto), reactive strength index (RSI), relative mean braking force ($\bar{F}$_brake/BM_), braking phase duration (T_Brake_), relative mean push-off power ($\bar{P}$_po/BM_) and relative peak power output (P_max/BM_).

For the DJ test, the significant PRE-POST changes are shown in Fig. 2. All absolute values are reported in the Supplementary file **S1**. All but 4 participants showed a decrease in vto and RSI. LSI analysis showed small increases during the braking phase (LSI-$\bar{F}$_Brake/BM_: from 4 ± 13 to 7 ± 14 %, p < 0.05, d = 0.58; LSI-F_max/BM_: from 1 ± 11 to 7 ± 12 %, p < 0.001, d = 0.57).

For the H-FvP test, the PRE-POST comparison showed only a small significant increase in the relative bilateral mean force ($\bar{F}$_/BM_) (Table 1). The LSI scores did not change significantly.

**Table 1:** Relative bilateral PRE-POST changes (mean ± SD in delta % PRE) in the H-FvP test.

*H-FvP*	Variables	delta %	Session	d
***Extrapolated parameters***	**R²**	2 ± 12	ns	
	$\bar{V}$_**0**_	10 ± 37	ns	
	$\bar{F}$_**0**_	2 ± 18	ns	
	$\bar{P}$_**max**_	7 ± 21	ns	
***Push-off phase***	**H**_**po**_	−3 ± 6	a	0.41
	**vto**	−1 ± 8	ns	
	$\bar{F}$_**/BM**_	3 ± 5	b	0.65
***Load 0 %***	**V**_**max**_	1 ± 8	ns	
***Load 60 %***	**F**_**max/BM**_	2 ± 5	ns	

d = Effect size measured with Cohen's d.

a p < 0.05.

b p < 0.01, ns: not significant.

Coefficient of determination (R^2^), extrapolated maximum velocity and force ($\bar{V}$_0_ and $\bar{F}$_0_), push-off distance (H_po_), take-off velocity (vto), normalised mean push-off force ($\bar{F}$_/BM_), peak velocity (V_max_) achieved at 0 % BM load and normalised peak force (F_max/BM_) at 60 % BM.

### 3.6 Principal component and clustering analyses

**Fig. 3:**
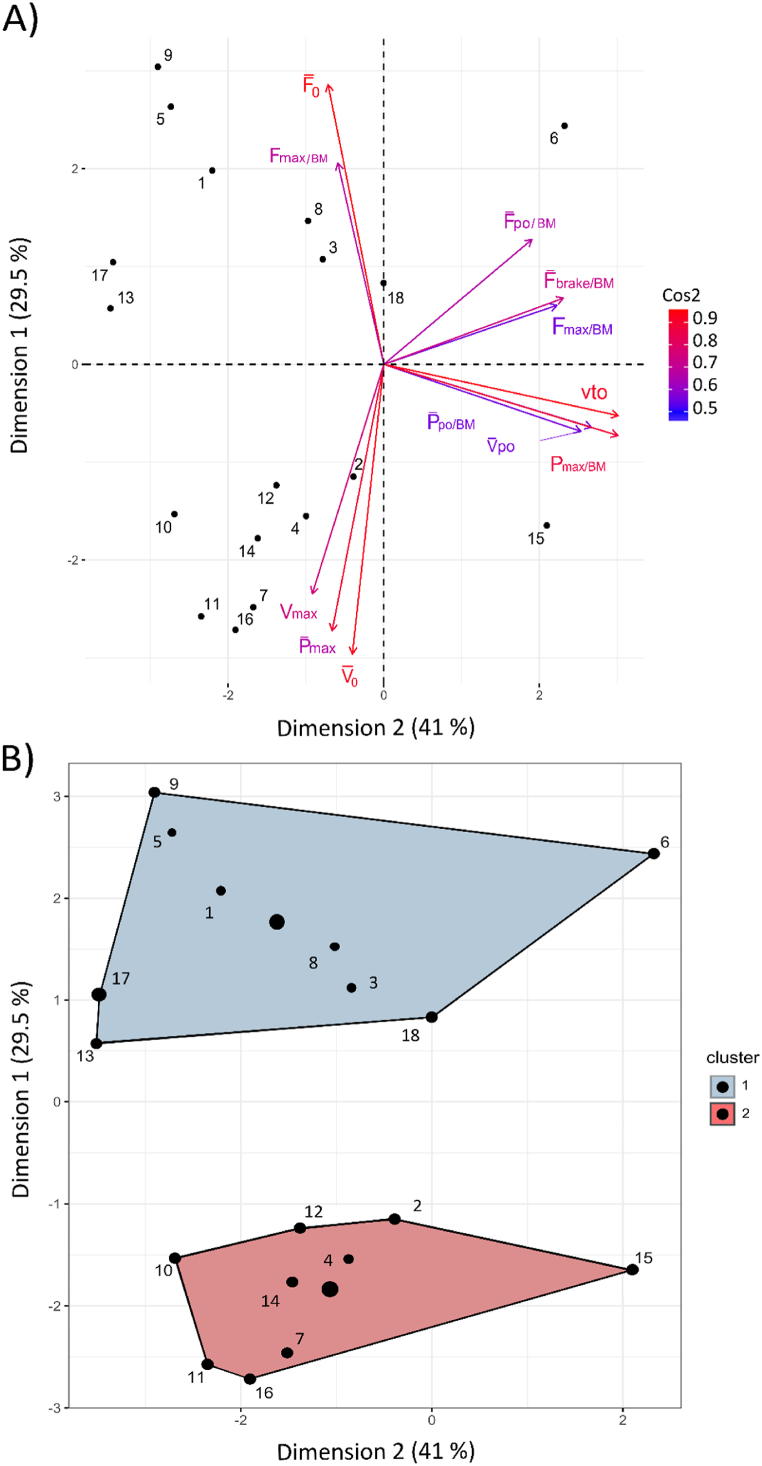
PCA and k-means analyses applied to the relative PRE-POST changes in the H-FvP and DJ tests. A) The axes represent the first two PCA dimensions. Participants are represented by a point and their coordinates represent their correlations with the two dimensions. B) The k-means method identified two clusters of 9 participants. The classification of participants within each cluster is represented by a concentration ellipse with a 95 % confidence interval.

The PRE-POST changes in the variables recorded in the H-FvP and DJ tests were significantly clustered according to the Hopkins test (H-value = 0.64). PCA analysis revealed that the first two dimensions accounted for 70 % of the variance in the data set (Fig. 3). The cos2 values were particularly high for the extrapolated variables ($\bar{F}$_0_ and $\bar{V}$_0_) of the H-FvP test and for the vto and P_max/BM_ values of the DJ test (Fig. 3A).

The k-means clustering method identified two distinct subgroups (Clusters 1 and 2) based on the PRE-POST changes in the H-FvP and DJ tests (Fig. 3B). The variables that contributed significantly (p < 0.001) to the subgroup separation were $\bar{F}$_0_, $\bar{V}$_0_ and $\bar{P}$_max_.

**Fig. 4:**
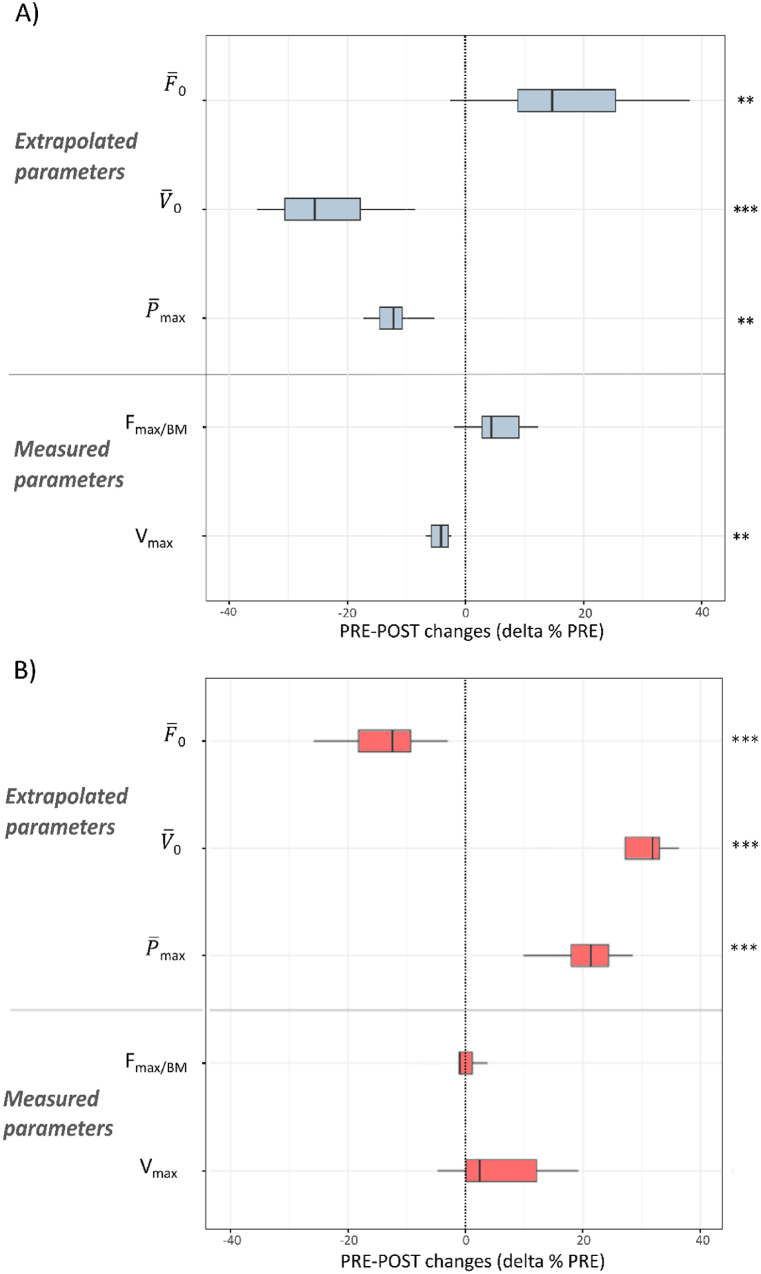
Relative PRE-POST changes (delta % PRE) for Clusters 1 (A) and 2 (B) in the H-FvP test. **p < 0.01, ***p < 0.001. Extrapolated maximum speed, force and power ($\bar{V}$_0_, $\bar{F}$_0_ and $\bar{P}$ max), normalised peak force (F_max/BM_) at 60 % BM and peak velocity (V_max_) achieved at 0 % BM loading.

For the H-FvP test, the relative PRE-POST changes in the extrapolated and measured parameters are detailed for each cluster in Fig. 4. Participants in Cluster 1 showed an increase in $\bar{F}$_0_ and a decrease in $\bar{V}$_0_ and $\bar{P}$_max_ values (Fig. 4A). Only participants in Cluster 1 showed a small decrease in V_max_ values. Conversely, Cluster 2 showed an increase in $\bar{P}$_max_ and $\bar{V}$_0_, and a decrease in $\bar{F}$_0_ (Fig. 4B). Only Cluster 2 showed an increase in LSI in $\bar{F}$_0_, from 3 ± 5 to 8 ± 7 % (p < 0.001), resulting from a decrease in LSI in $\bar{F}$_0_ of the dominant lower limb only.

**Fig. 5:**
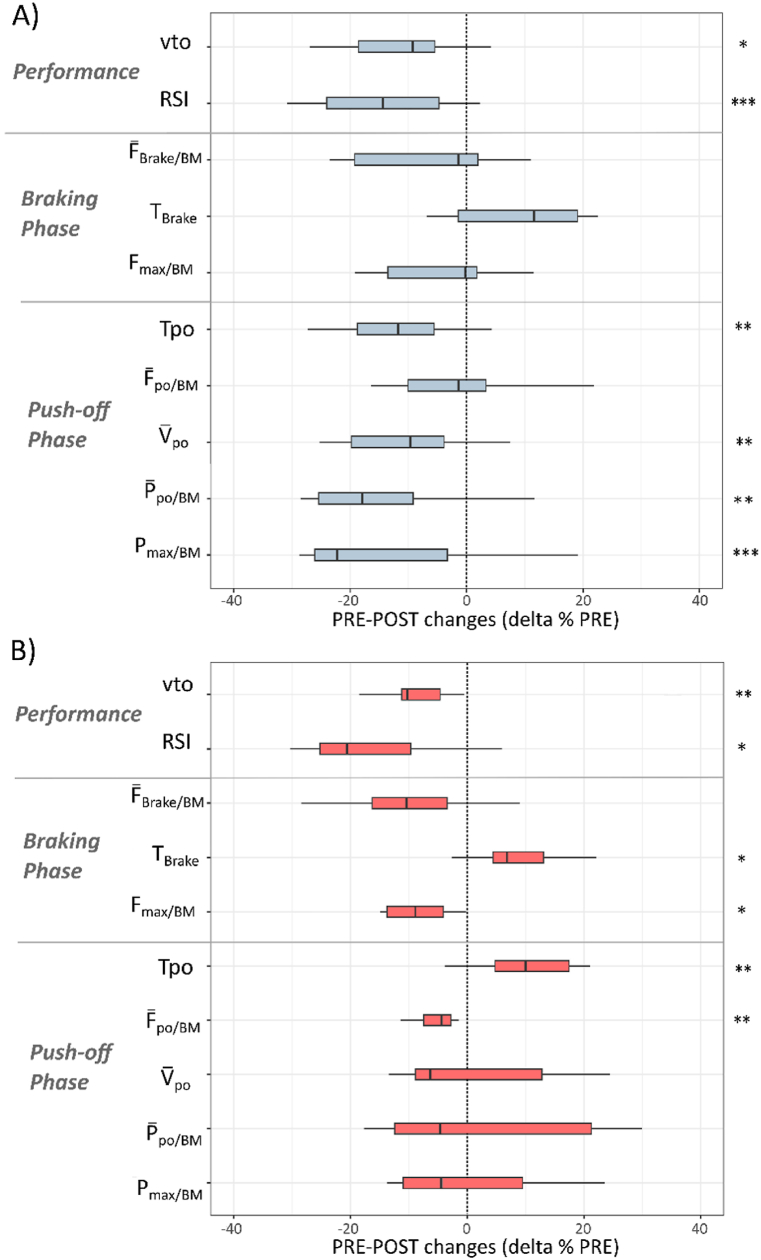
Relative PRE-POST changes (delta % PRE) for clusters 1 (A) and 2 (B) in the DJ test. *p < 0.05, **p < 0.01, ***p < 0.001. Take-off velocity (vto), reactive strength index (RSI); relative mean force ($\bar{F}$_brake/BM_), duration (T_Brake_) and relative peak force (F_max/BM_) of the braking phase; push-off phase duration (Tpo), relative mean force ($\bar{F}$_po/BM_), mean velocity ($\bar{V}$_po_), relative mean power and peak power output ($\bar{P}$_po/BM_, P_max/BM_).

For the DJ test, the relative PRE-POST changes for each cluster are shown in Fig. 5. A moderate decrease in performance (vto) and RSI was experienced by participants in each group. Those in Cluster 1 showed a moderate decrease in Tpo, and a large decrease in P_max/BM_, $\bar{V}$_po_ and $\bar{P}$_po/BM_ (Fig. 5A), whereas those in Cluster 2 showed a small increase in T_Brake_, a moderate increase in Tpo, and a moderate decrease in F_max/BM_ and $\bar{F}$_po/BM_ (Fig. 5B). Participants in Cluster 2 showed moderate to large increases in LSI for F_max/BM_ (from 1 ± 4 to 9 ± 5 %; p < 0.001; d = 0.7) and $\bar{F}$_Brake_/BM (from 1 ± 5 to 10 ± 6 %, p < 0.001; d = 1.2).

## 4 Discussion

To assess the individual fatigue effects of a 75-min football training session in elite young male players, a comprehensive battery of tests was used, including an H-FvP test and more classic metabolic measures, a 30 m sprint and a DJ test. Training loads and acute fatigue effects were close to those expected. In support of our first hypothesis, the standard football training session resulted in significant functional impairment, particularly in the DJ test, which also showed increased LSI and large inter-individual variability. Highlighting the interest and complementarity of the DJ and H-FvP tests, their combined analysis identified two subgroups of young players with specific functional changes with fatigue. These two subgroups were not otherwise different in terms of physical characteristics and total distance run during the football training session.

For the group as a whole, the training loads of the 75-min football training session - including total distance and high speed running distance - were comparable to those reported in similar cohorts [2,37]. In terms of acute fatigue effects, the measured metabolic changes were consistent with those reported after a simulated 90-min football match in 18-year-old players for pH (7.38 *vs.* 7.39), lactate (4.14 *vs*. 6.1 mmol L^−1^) and HCO_3_^−^ (21.58 *vs*. 21.73 mmol L^−1^) [38]. The reported subjective sensation of fatigue (6.2 ± 0.6) was also comparable to that reported in elite youth footballers after strenuous training, with a rating of 72 ± 16 on a scale of 0–100 ^2^. Finally, the observed 2 % slower time in the 30 m sprint test was close to the 2.4 % and 2 % reductions reported after the first and second halves of a football match [39]. Further confirming the suitability of this football training session to assess the acute fatigue effects experienced by players during regular training, the DJ test showed a significant 8 % reduction in vto. This is consistent with the 4.5 % reduction in DJ flight time reported in young footballers following a shorter 42-min football-specific intermittent session [40]. Clusters 1 and 2 did not differ significantly on any of these parameters.

Consistent with our first hypothesis, the DJ test revealed significant temporal and kinetic changes at POST, indicating functional deficits and an increased LSI. The observed small group mean increase in braking time and decrease in relative mean braking force are likely to indicate reduced resistance to ground impact and impaired stretch-shortening cycle performance [14]. In this line, most players showed a moderate decrease in RSI (−15 ± 20 %; from 0.54 ± 0.17 to 0.43 ± 0.19), which agrees with previous observations after a football match or simulated football match [18,41,42]. On the other hand, possibly due to a lack of familiarity with the DJ task [15] and/or the adoption of a compensatory ‘stiffer’ landing [43], 4 out of 18 players showed the opposite trend of an increase in RSI and vto performance. In order to improve routine testing during the football season, the kinetic DJ analysis should be implemented through kinematic recordings to better identify the individual compensatory strategies that could indicate certain players at risk [12]. Finally, the average 7 % increase in LSI for peak and mean braking force confirmed the sensitivity of interlimb asymmetry to fatigue in footballers, as reported in a single-leg counter-movement jump test [16]. This fatigue-induced increase was lower than the 15 % values reported in players with lower extremity injuries [9]. However, it is still noticeable in young footballers, who are more prone to training injuries than adults [10] and whose growth may already alter their landing mechanics and predispose them to injury [9]. These overall findings support the use of the DJ test and related RSI and LSI indices to detect functional deficits and potential injury risk under fatiguing conditions following a standard football training session. However, as expected, kinetic DJ analysis alone was not sufficient to understand the aetiology of the large inter-individual variability.

Confirming our second hypothesis, the analysis of the comined analysis of the DJ and H-FvP tests differentiated subgroups of young players with different types of functional changes with fatigue. These findings are consistent with the relevance of the FvP profile for the assessment of fatigue-related changes in the ability to generate force, velocity, and power during multi-joint ballistic tasks [25,44]. In the absence of fatigue, previous studies have shown that FvP profiles can be dominated by either force or velocity [45], and specific training programmes can be prescribed to reduce FvP imbalances [46]. In the case of fatigue, the FvP profile may also be useful in distinguishing athletes whose specific changes with fatigue would not be detected by conventional analyses of mean ground reaction force [25]. In the current study, highlighting the importance of the H-FvP test, the extrapolated values of $\bar{F}$_0_, $\bar{V}$_0_ and $\bar{P}$_max_ proved to be valuable in identifying two subgroups of players with specific fatigue-induced changes. Despite a different range of the highest velocities achieved at PRE in DJ ($\bar{V}$_po_: 1.05–1.40 m s^−1^) and estimated in the H-FvP test ($\bar{V}$_0_: 2.45–5.14 m s^−1^), the functional changes with fatigue were similar in both tests, but opposite between the two subgroups: Cluster 1 showed a decrease in $\bar{V}$_0_ and $\bar{P}$_max_ in the H-FvP test, and in mean velocity, mean relative power, and maximum power in the DJ test. Conversely, Cluster 2 showed a decrease in $\bar{F}$_0_ in the H-FvP test, and a decrease in the mean relative push-off force in the DJ test. Furthermore, although both clusters exhibited a decline in DJ performance, they showed contrasting alterations in Tpo, which is likely to account for the majority of the kinetic changes: In Cluster 1, the decrease in Tpo with fatigue was associated with a decline in mean push-off velocity and velocity at take-off. In the H-FvP test, the observed decrease in $\bar{V}$_0_ and $\bar{P}$_max_ with fatigue suggests that these players were less efficient in push-off production as velocity increased. Nevertheless, in the absence of joint kinematics recordings, it cannot be excluded that these players adopted a relatively stiffer ankle joint once fatigued [43]. Conversely, for Cluster 2, which showed a decrease in $\bar{F}$_0_ in the H-FvP test, the observed increase in both T_Brake_ and Tpo in DJ may have compensated for their decreased ability to generate force, allowing them to maintain a similar force integral and mean velocity. Finally, only Cluster 2 demonstrated an increased asymmetry with fatigue in maximal and mean braking force in DJ. As previously reported [9], asymmetry during the landing phase is a significant predictor of injury in elite young footballers, with a 10 % increase in injury risk for each percentage increase in asymmetry. However, at this stage, the current study is unable to identify the underlying structural and neural mechanisms that may have contributed to the cluster-specific changes observed with fatigue.

### 4.1 Strengths and limitations

The strength of our study lies in the analysis of young elite footballers, a cohort characterised by high variability in performance and susceptibility to injury, which plays a crucial role in the development of functional qualities. Based on the complementarity of the results obtained from the DJ and the H-FvP, the cluster analysis revealed two distinct fatigue profiles that were not apparent from the independent analysis of each test alone. However, methodological limitations should be taken into account: (i) the delay of the POST test undoubtedly attenuated the acute effects of fatigue; (ii) despite clear instructions and an exclusive selection of correctly performed DJ tests, a lack of familiarity was noted in some of these young players, highlighting the need to familiarise athletes prior to such field testing; (ii) due to the turnover of players at the training centre, there was no monitoring of their performance and injuries over the next few months.

## 5 Practical applications

The results of this study may prove instructive to practitioners when selecting performance tests and metrics to gain insight into a player's level of readiness and fatigue. The current test battery was found to be useful for coaches and medical staff to individualise their interventions with this young population of footballers. It would be worthwhile to repeat it throughout the season to help identify and monitor individual fatigue-induced deficits and potential risk factors.

## 6 Conclusion

The test battery was able to differentiate between players according to their specific fatigue-induced changes in the H-FvP relationship and force production in the DJ test. The extrapolated values from the H-FvP test allowed the identification of two subgroups with opposite fatigue profiles in terms of force and velocity changes. In one of these subgroups, the DJ kinetic changes indicated a potential risk of injury due to increased interlimb asymmetry with fatigue. This comprehensive test battery highlights its value in improving the individual monitoring of young footballers. However, future research is needed (i) to compare the results of the laboratory H-FvP test with those of a field sprint FvP test and (ii) to follow a larger number of players longitudinally throughout the season, particularly those identified as at risk by the test battery, with and without subsequent individualised staff intervention.

## Declarations

### 6.1 Ethics statement

#### 6.1.1 Human experiments

This study was conducted in accordance with the Helsinki Convention and was approved by the CERSTAPS National Ethics Committee, approval number IRB00012476-2021-13-04-106, entitled “Combining a battery of tests and a mathematical model to improve injury prevention in young semi-professional footballers”. All participants were informed of the aims of the research, the procedures involved and the potential risks. Written informed consent was obtained from each participant prior to inclusion in the study. Personal data were kept anonymous and confidential to ensure that participants' privacy was protected in accordance with data protection standards.

## Fundings

This research did not receive any specific funding.

## Additional information

No additional information is available for this paper.

## Data availability statement

Data will be made available on request.

## CRediT authorship contribution statement

**Adrien Mangini:** Writing – original draft, Methodology, Data curation, Conceptualization. **Robin Macchi:** Writing – review & editing. **Dorian Giraud:** Writing – review & editing. **Laura Pomportes:** Supervision. **Paul Galantine:** Writing – review & editing. **Denis Bertin:** Project administration. **Caroline Nicol:** Writing – review & editing, Supervision. **Arnaud Hays:** Writing – review & editing, Writing – original draft, Validation, Project administration.

## Declaration of competing interest

The authors declare that they have no known competing financial interests or personal relationships that could have appeared to influence the work reported in this paper.
