# Supplementary material for: Towards a more individualised assessment of post-training fatigue in young footballers
Source: Heliyon. 2024 Jun 27;10(13):e33626. doi: 10.1016/j.heliyon.2024.e33626 (PMC11255448; doi:10.1016/j.heliyon.2024.e33626)

**Table S1****.** Mean values, relative PRE-POST changes (in delta % PRE) and LSI (%) for the DJ test. *d = Effect size measured with Cohen's d. *p < 0.05, **p<0.01, ns: not significant.*

| ***DJ*** | **Variables** |  | **PRE** | **POST** | **delta %** | **Session** | **d** |
| --- | --- | --- | --- | --- | --- | --- | --- |
| ***Performance*** | **vto** | *BLT* | 1.91 ± 0.30 | 1.75 ± 0.30 | -8 ± 9 | * | 0.82 |
|  | **RSI** | *BLT* | 0.54 ± 0.17 | 0.43 ± 0.19 | -15 ± 20 | * | 0.72 |
| ***Braking***  ***phase*** | **F̅_brake/BM_** | *BLT* | 34.1 ± 12.7 | 30.9 ± 10.8 | -8 ± 12 | ** | 0.53 |
|  |  | *LSI (%)* | 4 ± 13 | 7 ± 14 |  | * | 0.58 |
|  | **T_Brake_** | *BLT* | 0.36 ± 0.12 | 0.38 ± 0.12 | 8 ± 9 | * | 0.64 |
|  | **F_max/BM_** | *BLT* | 34.1 ± 12.7 | 31.24 ± 10.6 | - 7 ± 11 | ns |  |
|  |  | *LSI (%)* | 9 ± 8 | 9 ± 9 |  | ** | 0.57 |
| ***Push-off phase*** | **T_po_** | *BLT* | 0.19 ± 0.03 | 0.18 ± 0.05 | -1 ± 7 | ns |  |
|  | **F̅_po/BM_** | *BLT* | 21.2 ± 3.5 | 20.3 ± 3.2 | -4 ± 7 | ns |  |
|  |  | *LSI (%)* | 2 ± 8 | 5 ± 7 |  | ns |  |
|  | $\overline{\mathbf{V}}$**_po_** | *BLT* | 1.26 ± 0.14 | 1.15 ± 0.18 | -6 ± 11 | ns |  |
|  | $\overline{\mathbf{P}}$**_po/BM_** | *BLT* | 1293.1 ± 377.4 | 1203.7 ± 473.2 | -7 ± 17 | * | 0.33 |
|  |  | *LSI (%)* | 1 ± 12 | 2 ± 11 |  | ns |  |
|  | **P_max/BM_** | *BLT* | 2 ± 11 | 34.9 ± 8.6 | -8 ± 17 | * | 0.46 |
|  |  | *LSI (%)* | 1 ± 8 | 1 ± 8 |  | ns |  |

**Table S2.** Mean values, relative PRE-POST changes (in delta % PRE) and LSI (%) for the H-FvP test*. d = Effect size measured with Cohen's d. *p < 0.05, ns: not significant.*

| ***H-FvP*** | **Variables** |  | **PRE** | **POST** | **delta %** | **Session** | **d** |
| --- | --- | --- | --- | --- | --- | --- | --- |
| ***Extrapolated variables*** | **R²** | *BLT* | 0.79 ± 0.15 | 0.81 ± 0.17 | 2 ± 12 | ns |  |
|  | $\overline{\mathbf{V}}$**_0_** | *BLT* | 3.69 ± 1.11 | 3.87 ± 1.09 | 10 ± 37 | ns |  |
|  |  | *LSI (%)* | 3 ± 8 | 9 ± 11 |  | ns |  |
|  | $\overline{\mathbf{F}}$**_0_** | *BLT* | 1384 ± 283 | 1396 ± 331 | 2 ± 18 | ns |  |
|  |  | *LSI (%)* | 4 ± 11 | 1 ± 8 |  | ns |  |
|  | $\bar{\mathbf{P}}$**_max_** | *BLT* | 1277 ± 480 | 1331 ± 439 | 7 ± 21 | ns |  |
|  |  | *LSI (%)* | 2 ± 11 | 12 ± 14 |  | ns |  |
| ***Push-off phase*** | **Hpo** | *BLT* | 0.41 ± 0.05 | 0.39 ± 0.05 | -3 ± 6 | ns |  |
|  | **vto** | *BLT* | 2.00 ± 0.31 | 1.98 ± 0.29 | -1 ± 8 | ns |  |
|  | $\overline{\mathbf{F}}$**_/BM_** | *BLT* | 19.50 ± 2.86 | 20.18 ± 2.14 | 3 ± 5 | ** | 0.26 |
|  |  | *LSI (%)* | 9 ± 8 | 9 ± 9 |  | ns |  |
| ***Load 0%*** | **V_max_** | *BLT* | 1.28 ± 0.15 | 1.28 ± 0.16 | 1 ± 8 | ns |  |
| ***Load 60%*** | **F_max/BM_** | *BLT* | 16.8 ± 2.0 | 17.22 ± 2.23 | 2 ± 5 | ns |  |
|  |  | *LSI (%)* | 5 ± 7 | 4 ± 8 |  | ns |  |


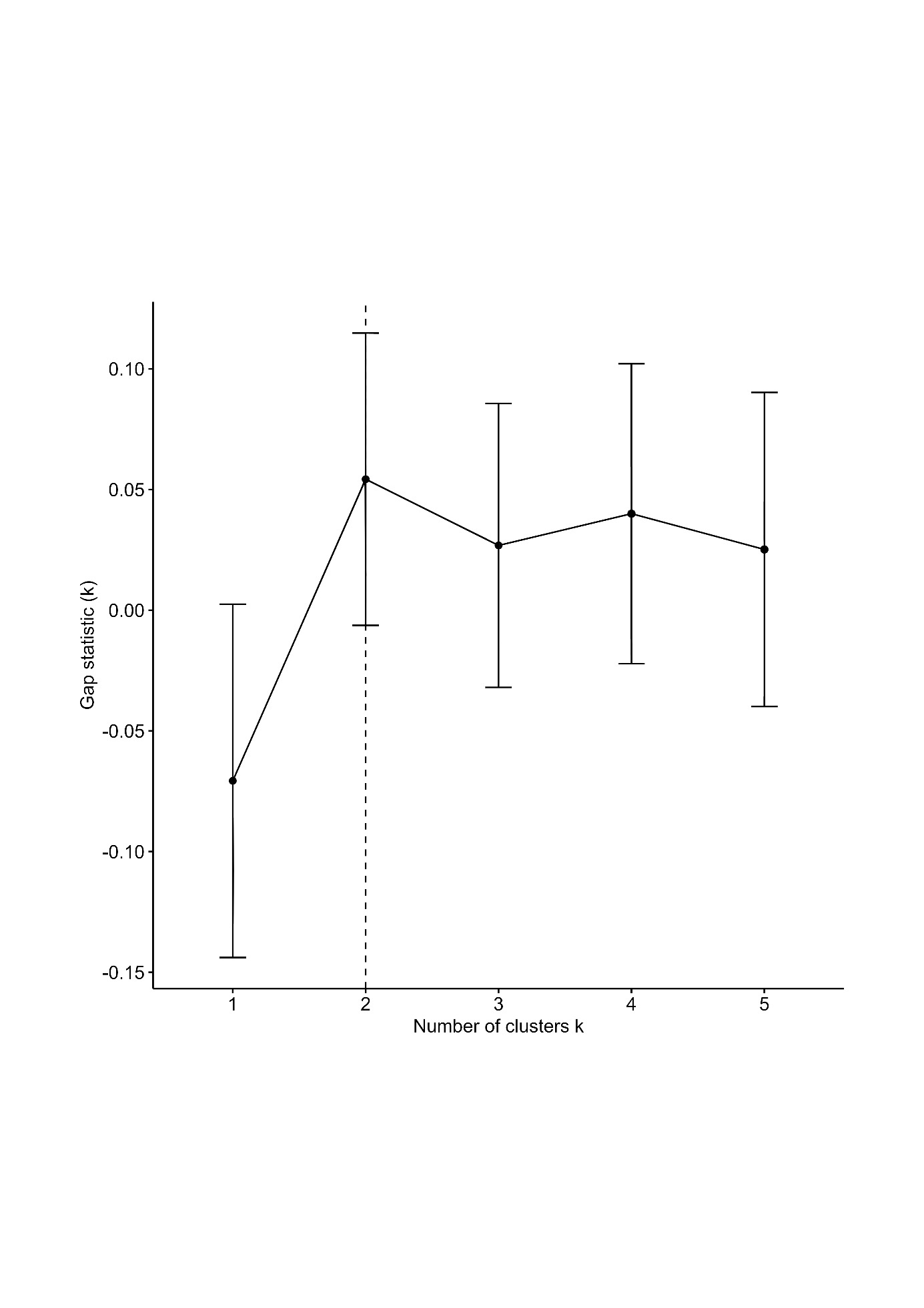


**Figure S1.** Gap statistic method applied to the data set obtained from the H-FV and DJ tests. *d = Effect size measured with Cohen's d. *p < 0.05, **p<0.01, ***p<0.001, ns: not significant.*

**Table S3.** Table of results of the analysis of variance (ANOVA) for the DJ test (n=18). **p < 0.05, **p<0.01..*

*
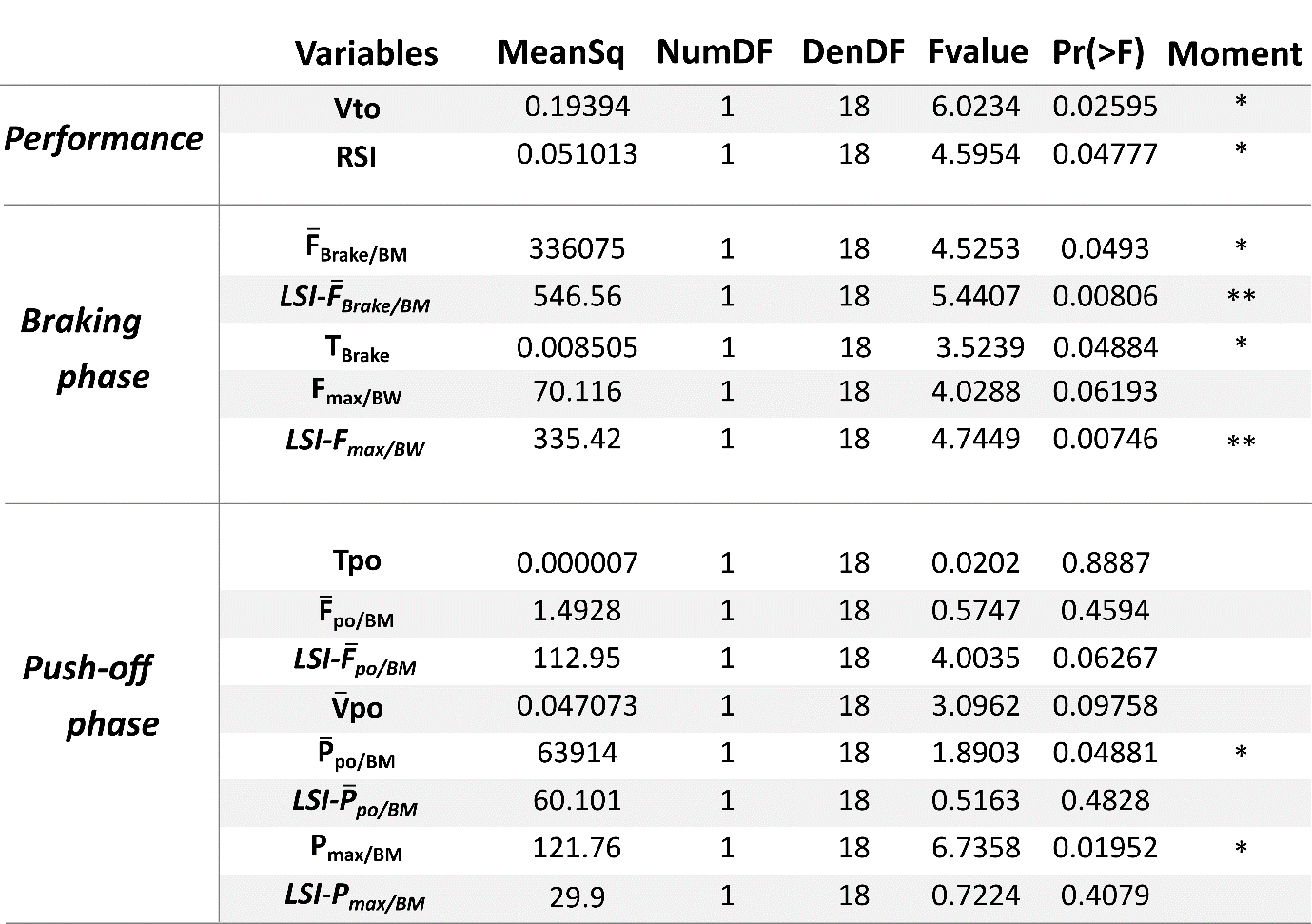
*

**Table S4.** Table of results of the analysis of variance (ANOVA) for the H-FvP test (n=18)*. *p < 0.05, **p<0.01.*

*
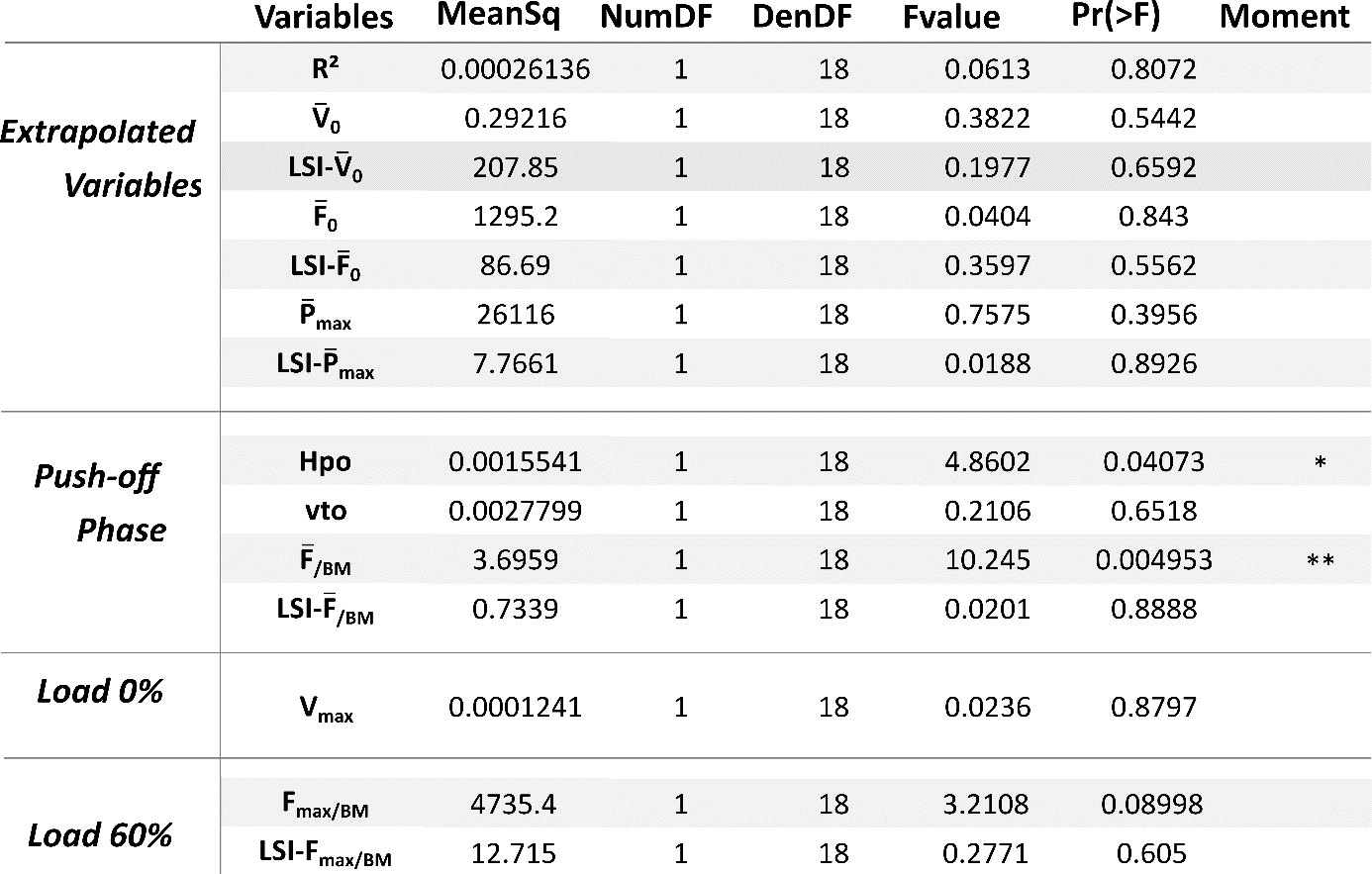
*

**Table S5.** Table of results of the analysis of variance (ANOVA) for Cluster 1 (n=9) of the DJ test*. *p < 0.05, **p<0.01, ***p<0.001.*


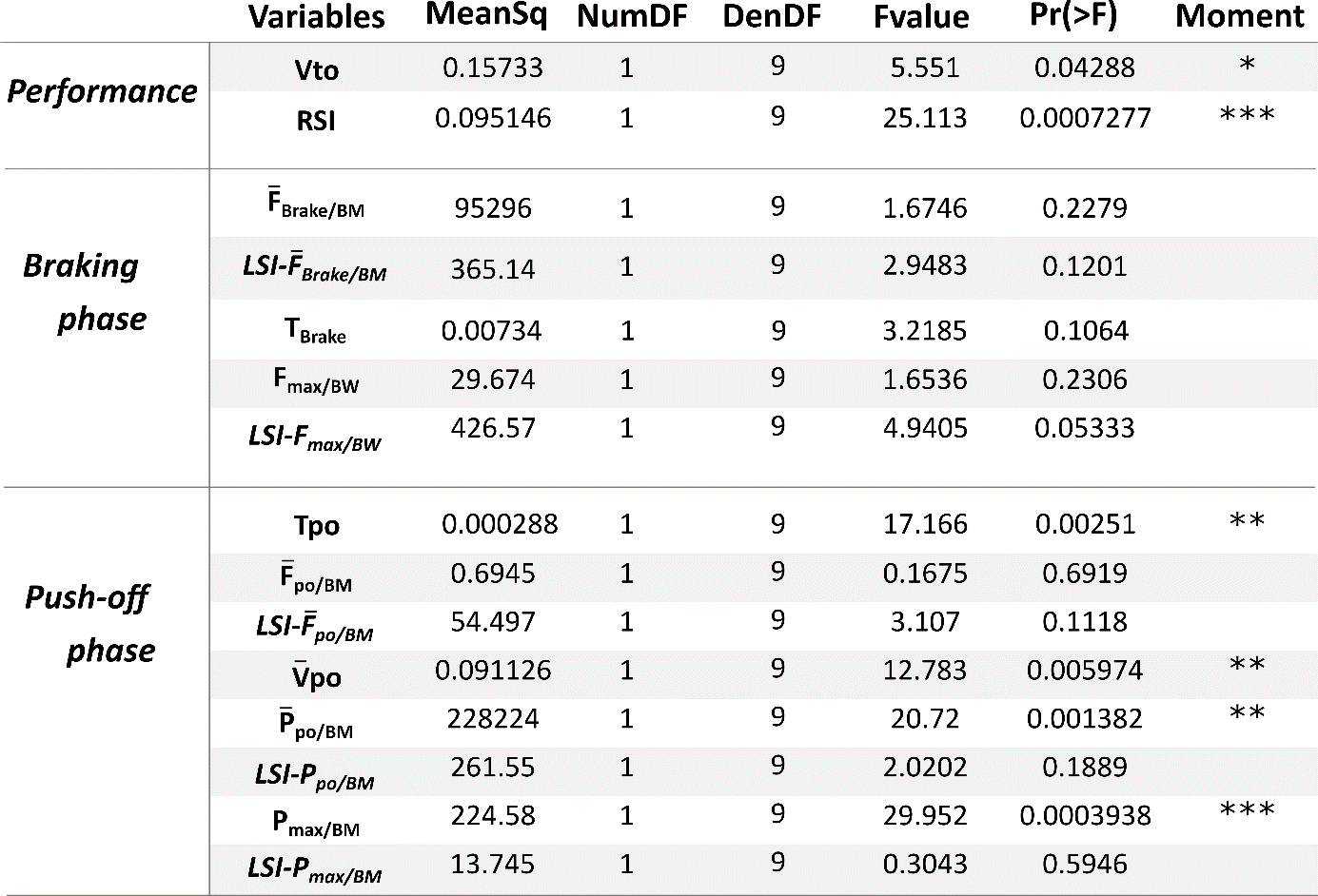


**Table S6.** Table of results of the analysis of variance (ANOVA) for Cluster 1 (n=9) of the H-FvP test*. *p < 0.05, **p<0.01, ***p<0.001.*


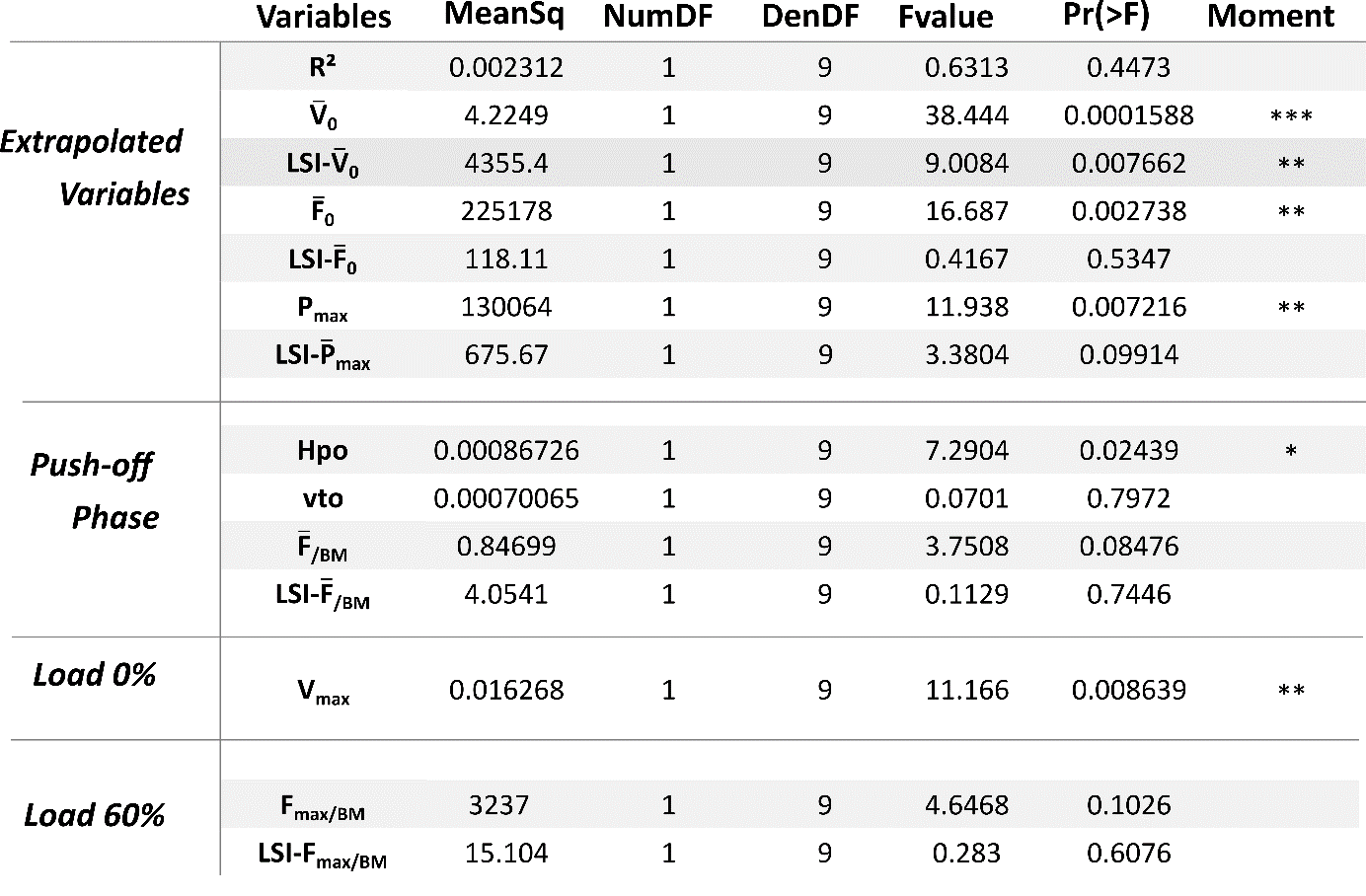


**Table S7.** Table of results of the analysis of variance (ANOVA) for Cluster 2 (n=9) of the DJ test*. *p < 0.05, **p<0.01.*

**
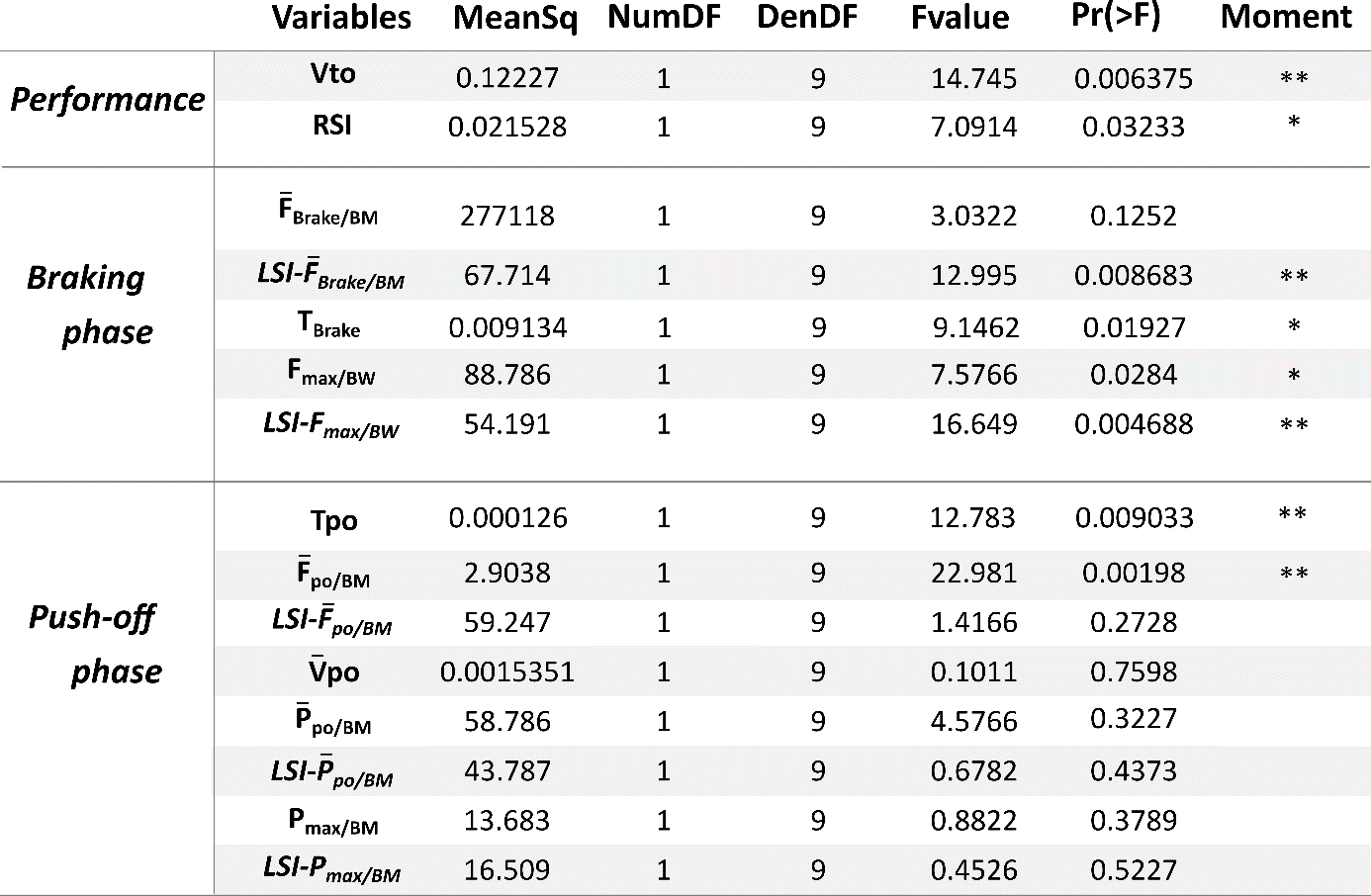
**

**Table S8.** Table of results of the analysis of variance (ANOVA) for Cluster 2 (n=9) of the H-FvP test*. *p < 0.05, ***p<0.001.*


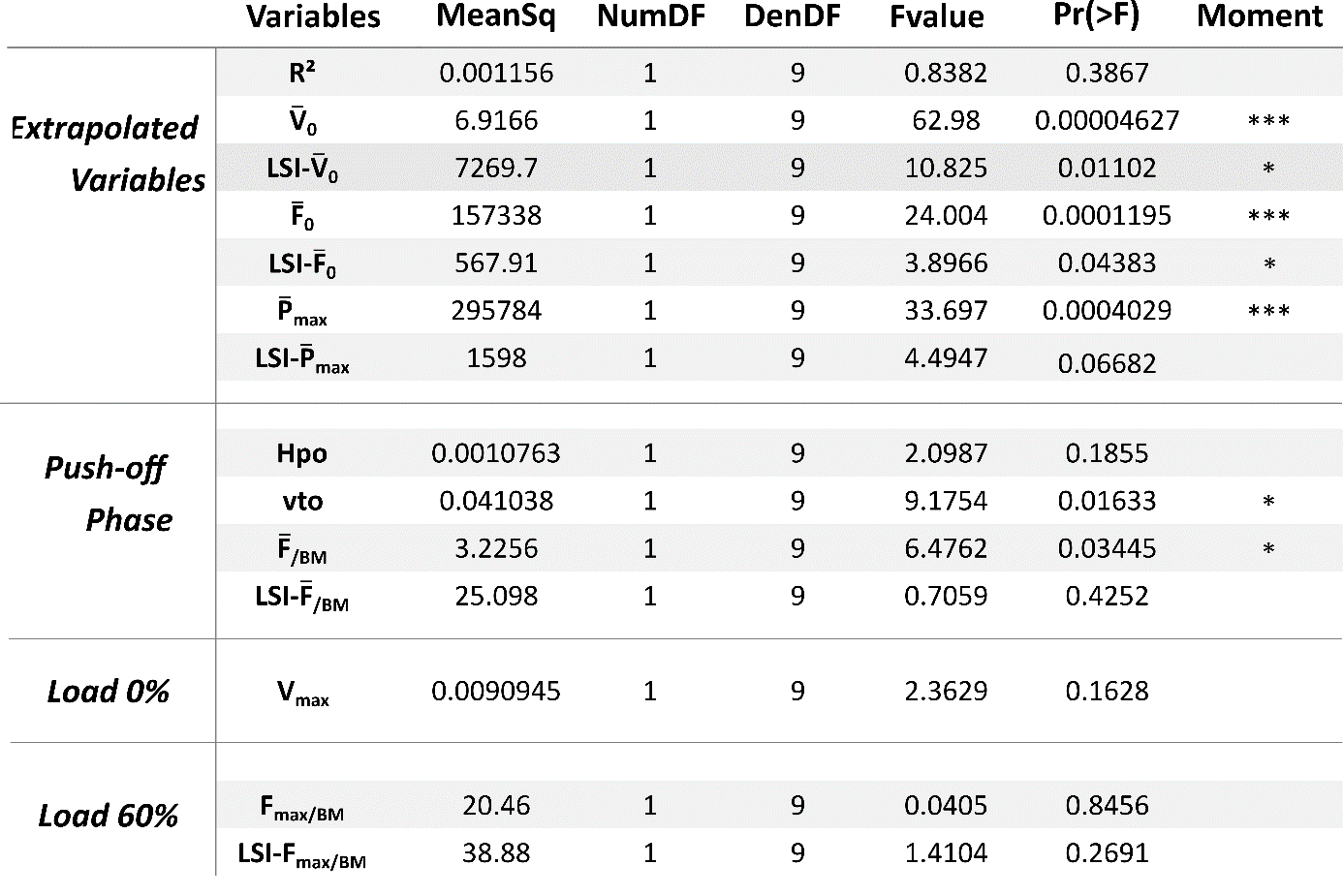

Supplement: Multimedia component 1 [file mmc1.docx]
